# Facilitating EMA binding test performance using fluorescent beads combined with next‐generation sequencing

**DOI:** 10.1002/jha2.277

**Published:** 2021-09-09

**Authors:** Andreas Glenthøj, Christian Brieghel, Amina Nardo‐Marino, Richard van Wijk, Henrik Birgens, Jesper Petersen

**Affiliations:** ^1^ Centre for Haemoglobinopathies Department of Haematology Rigshospitalet Copenhagen University Hospital Copenhagen Denmark; ^2^ Central Diagnostic Laboratory‐Research University Medical Center Utrecht Utrecht University Utrecht The Netherlands

**Keywords:** haemolytic anaemia, hereditary anaemias, laboratory haematology, rbc membrane, spherocytosis

## Abstract

The eosin‐5′‐maleimide (EMA) binding test is widely used as diagnostic test for hereditary spherocytosis (HS), one of the most common haemolytic disorders in Caucasian populations. We recently described the advantages of replacing the use of healthy control blood samples with fluorescent beads in a modified EMA binding assay. In this study we further explore this novel approach. We performed targeted next‐generation sequencing, modified EMA binding test and osmotic gradient ektacytometry on consecutive individuals referred to our laboratory on the suspicion of HS. In total, 33 of 95 carried a (likely) pathogenic variant, and 24 had variants of uncertain significance (VUS). We identified a total 79 different (likely) pathogenic variants and VUS, including 43 novel mutations. Discarding VUS and recessive mutations in *STPA1*, we used the occurrence of (likely) pathogenic variants to generate a diagnostic threshold for our modified EMA binding test. Twenty‐one of 23 individuals with non‐*SPTA1* (likely) pathogenic variants had EMA ≥ 43.6 AU, which was the optimal threshold in receiver operating characteristic (ROC) analysis. Accuracy was excellent at 93.4% and close to that of osmotic gradient ektacytometry (98.7%). In conclusion, we were able to simplify the EMA‐binding test by using rainbow beads as reference and (likely) pathogenic variants to define an accurate cut‐off value.

## 1 INTRODUCTION

Hereditary spherocytosis (HS) is a relatively common and well‐characterised hereditary haemolytic disorder. The disease is particularly common in people of northern European descent, with a prevalence of approximately 1:2000 in this population [1, 2]. The genetic background of HS is germline mutations in red blood cell (RBC) cytoskeleton proteins, such as α‐spectrin, β‐spectrin, band 3 and ankyrin [3, 4].

Individuals with HS typically present with Coombs‐negative haemolytic anaemia, high MCHC and splenomegaly. In many cases, however, clinical features and paraclinical findings are equivocal and advanced laboratory tests are necessary to confirm the diagnosis.

The Eosin 5‐Maleimide (EMA) binding test is recommended as the primary screening test for HS, both sensitivity and specificity of this method being over 90% [1]. In this test RBCs are incubated with EMA, which binds extracellular membrane‐associated proteins. EMA fluorescence can be detected by flowcytometry and mainly reflects decreased RBC Band 3, which in HS is reduced compared to healthy controls [2]. Given its simplicity and the wide availability of flow cytometers, this test can be employed in most laboratories at a low cost. Often, results are reported as a ratio of the individual's mean fluorescent intensity (MFI) to that of healthy controls, making the test somewhat comparable across laboratories [5]. This approach does, however, require blood samples from up to six healthy – and ideally age matched ‐ controls, which can be challenging to locate [5, 6, 7]. We recently described a modified version of the EMA binding test, in which we substituted healthy control samples with fluorescent beads. [8]. Although healthy controls were still utilised for calibration, the number of control samples needed was reduced significantly. Performance of this modified EMA binding test was compared to that of the traditional method, using osmotic gradient ektacytometry as validation. We found that accuracy was not compromised, making this approach an attractive and simple alternative [8].

Osmotic gradient ektacytometry is a method for determining RBC deformability and is increasingly used due to the advent of a new generation of ektacytometers [9, 10]. Although this test reliably identifies the RBC characteristics associated with HS, it is incapable of discriminating spherocytes in HS from autoimmune haemolytic anaemia [10].

To facilitate HS diagnosis, targeted next‐generation sequencing (tNGS) is used to detect germline mutations in genes encoding for RBC cytoskeleton proteins [11, 12, 13, 14, 15, 16, 17, 18, 19, 20, 21]. tNGS is less time consuming than traditional sequencing techniques, but the technique is associated with high costs and long turnaround. tNGS can be an advantageous diagnostic tool, particularly in transfused individuals where functional testing is affected by donor blood. Nevertheless, the accuracy of tNGS alone has proven somewhat limited for the diagnosis of hereditary anaemias [11, 12, 14, 16–19, 21–32].

Results from the EMA binding test and osmotic gradient ektacytometry are often sufficient to diagnose HS, but both tests have limitations and may produce equivocal results [33, 34, 35]. Many previous studies have evaluated these tests mainly using clinical features of HS as proof of disease, hereby creating an inherent risk of confirmation bias [36].

In this study, we wish to further investigate the modified EMA binding test using rainbow beads instead of healthy control samples. By defining HS as the presence of diagnostic cytoskeleton protein gene mutations identified using tNGS and validating results using osmotic gradient ektacytometry, we provide a reproducible way of estimating a cut‐off value for the modified EMA‐binding test. Finally, we briefly describe the identified underlying pathogenic mutations.

## 2 MATERIALS AND METHODS

### 2.1 Population

**FIGURE 1:**
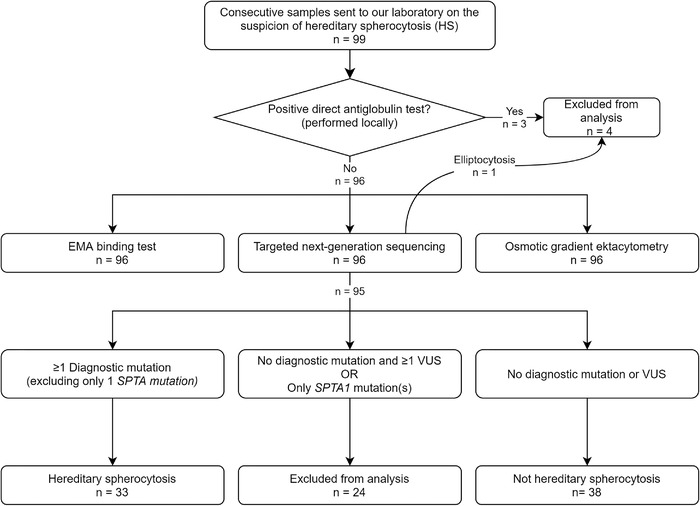
**Study flowchart**. Samples were subjected to modified EMA‐binding test, osmotic gradient ektacytometry and targeted next‐generation sequencing. Individuals with a positive Coombs test, only *SPTA1* mutations, elliptocytosis or variants of uncertain significance (VUS) without a (likely) pathogenic variant were excluded from analysis of the EMA‐binding test threshold value

We included samples from all individuals referred to our laboratory with suspected HS between 1st May 2017 and 1st July 2018 (Figure 1). As samples were shipped from other institutions, clinical data were not available. Samples have previously been used to test the performance of the EMA binding test using fluorescent beads versus healthy controls [8].

### 2.2 Ethics

Data were stored and handled in accordance with permission from the Danish Data Protection Agency (10122009 HEH‐L.HB). All participants or a parent/guardian consented to diagnostic tests for haemolytic anaemia including tests for HS.

### 2.3 tNGS

Genomic DNA was extracted from peripheral blood using the QIAamp DNA Blood Mini QIAcube Kit (Qiagen, Hilden, Germany) according to the manufacturer′s instructions. We used a small panel targeting RBC disorders, including genes covering the cytoskeleton proteins, *SPTA1* (α‐spectrin), *SPTB* (β‐spectrin), *ANK1* (ankyrin 1), *SCL4A1* (band 3), *EPB41* (protein 4.1) and *EPB42* (protein 4.2). Targeting, amplification and normalisation was performed according to the manufacturer′s instructions (TruSeq Custom Amplicon v1.5, Illumina, CA, USA). Sequencing was performed on a MiniSEquation (Illumina) via MiniSeq Mid Output Kit (300x paired‐end; Illumina). Sequencing analyses were performed using BaseSpace Variant Interpreter (Illumina) and Integrative Genomics Viewer software [37]. Variants were called with at least 10 variant reads, a minimum read depth of 30x and classified in categories according to recommendations from the American College of Medical Genetics and Genomics [38] using BaseSpace Variant Interpreter (Illumina). Only variants classified as likely pathogenic or pathogenic, referred to as (likely) pathogenic variants, and variants of uncertain significance (VUS) were included in the analyses. Benign mutation and likely benign mutations were excluded. Variants not previously described in ClinVar, dbSNP or COSMIC according to Alamut Visual (Interactive Biosoftware, Rouen, France) were reported as novel mutations.

### 2.4 EMA binding test

The EMA binding test was performed on EDTA‐stabilised blood within 48 h of sampling. The labeling of RBCs with EMA, usage of mid‐range FL1 Rainbow Fluorescent Particles (BD Biosciences, NJ, USA) and flow cytometry was performed as previously described [8]. A detailed protocol is available online (s). Sample evaluation was performed as a ratio comparison between the MFI of beads and patient. The ratio was calculated as the ΔMFI % (mean fluorescence intensity) using ([MFI_Rainbow beads_ ‐ MFI_Patient_]/MFI_Rainbow beads_) × 100.

$$\mathrm{EMA}\;=\;\Delta MFI\left(\%\right)\;=\left(1-\frac{MFI_{patient}}{MFI_{rainbow}\;\times \;CF}\right)\;\times \;100\%$$

A correction factor was used to adjust the MFI of new rainbow bead MFI lots, compared to the previous lots. To minimise the risk of bead MFI fluctuations, we used a second type of beads as control (FluoroSpheres K0110, Agilent Technologies Denmark ApS, Glostrup, Denmark), assuming the MFI ratio between these two sets of beads would remain constant.

### 2.5 Osmotic gradient ektacytometry

Osmotic gradient ektacytometry was performed on EDTA stabilised blood within 48 h of sampling, using a LoRRca ektacytometer (RR Mechatronics, Zwaag, Netherlands) as previously described [9]. Two parameters were evaluated on the ektacytometry curve: O_min_ and EI_max_. O_min_ reflects the minimal RBC surface/volume ratio, increasing in conditions with reduced surface/volume ratio such as HS [39]. EI_max_ reflects the maximal deformability of the RBCs. Reduction of EI_max_ typically represents a reduced RBC surface area, as is seen in HS [39]. O_hyper_, which reflects hydration status, was not used in this setting as this has been found either high or low in HS [40].

### 2.6 Statistical analyses

Statistical analyses were performed in ‘R’ version 3.6.3 [41] using packages ggplot2, caret, and pROC.

## 3 RESULTS

### 3.1 Population

A total of 99 individuals were included in the study. Fifty‐six (56%) were female and the mean age was 30.7 years (SD 28.3). Three individuals had a positive Coombs test (HS9, HS14 and HS33) and were excluded from further analyses (Figure 1) leaving 96 individuals for further analyses. None of the three Coombs positive individuals had (likely) pathogenic variants.

### 3.2 Mutations identified

Excluding 26 variants classified as likely benign, we identified a total of 78 variants in 58 of the 96 individuals (Table 1). Of these, 34 were (likely) pathogenic variants and 43 were VUS. Mutations in *SPTA1* and *SPTB* were predominant (Tables I and II). Apart from three intronic mutations (two single nucleotide substitutions and one deletion), all VUS were missense mutations (93%). In contrast, 32 of the 34 (likely) pathogenic variants (94%) were non‐missense mutations. To our knowledge, 42 mutations (26 (likely) pathogenic variants and 16 VUS) had not previously been described and, thus, were regarded as novel mutations (Table 1). One individual ('HS34') carrying a pathogenic *EPB41* mutation was excluded from further analyses, as examination of a peripheral blood smear confirmed the diagnosis of hereditary elliptocytosis (Figure 1). Forty‐one patients harbored the common *SPTA1* mutation c.6531‐12C > T (α‐spectrin^LELY^), which is considered benign in itself but may cause overt HS, hereditary elliptocytosis or hereditary pyropoikilocytosis in trans to *SPTA1* mutations [42, 43].

**TABLE 1:** Specification of mutations in the red blood cell cytoskeleton protein genes (SCL4A1, SPTB, SPTA1, ANK1, EPB41 and EPB42) in 99 patients with suspected hereditary spherocytosis

ID	Gene ID	cDNA	Protein change	Exon	Classification	Zygosity	Translation impact	Novel	EMA	O_min_	EI_max_
HS1	*SLC4A1*	c.118G > A	p.(Glu40Lys)	Exon 4	Likely benign*	Htz	missense	No	55.2	192	0.506
	*SPTB*	c.5290G > T	p.(Glu1764*)	Exon 25	Likely pathogenic	Htz	nonsense	Yes			
	*SPTA1*	c.6549‐4C > G	NA	Intron 46	VUS	Htz	intronic	No			
HS2	*SLC4A1*	c.1890+1G > T	Splice site	Intron 15	Likely pathogenic	Htz	splice	Yes	44.9	179	0.553
HS3	*SPTB*	c.1515delT	p.(Asn505Lysfs*68)	Exon 11	Likely pathogenic	Htz	frameshift	Yes	53.4	170	0.539
HS4	*SPTB*	c.398T > G	p.(Met133Arg)	Exon 3	VUS	Htz	missense	Yes	42.2	189	0.573
HS5	*SLC4A1*	c.118G > A	p.(Glu40Lys)	Exon 4	Likely benign*	Htz	missense	No	49.6	194	0.531
	*SPTB*	c.145dupG	p.(Ala49Glyfs*3)	Exon 1	Likely pathogenic	Htz	frameshift	Yes			
HS6	*SPTA1*	c.2909C > A	p.(Ala970Asp)	Exon 21	Likely benign*	Htz	missense	No	51.7	179	0.481
	*ANK1*	c.5224C > T	p.(Gln1742*)	Exon 40	Likely pathogenic	Htz	nonsense	Yes			
HS7	*SPTA1*	c.4605+1G > A	Splice site	Intron 32	Likely pathogenic	Htz	splice	Yes	42.6	168	0.566
	*EPB41*	c.1700G > A	p.(Gly567Asp)	Exon 12	VUS	Htz	missense	No			
	*SPTA1*	c.6896G > T	p.(Cys2299Phe)	Exon 50	VUS	Htz	missense	No			
	*SPTA1*	c.6531‐12G > A	NA	Intron 45	Likely benign*	Htz	intronic	No			
HS8	*SPTB*	c.3764+1G > A	Splice site	Intron 16	Likely pathogenic	Htz	splice	Yes	48.7	183	0.542
	*SPTA1*	c.2909C > A	p.(Ala970Asp)	Exon 21	Likely benign*	Htz	missense	No			
HS10	*SLC4A1*	c.1030C > T	p.(Arg344*)	Exon 10	Pathogenic	Htz	nonsense	No	48.9	198	0.538
HS11	*SLC4A1*	c.1030C > T	p.(Arg344*)	Exon 10	Likely pathogenic	Htz	nonsense	No	49.0	200	0.524
	*SPTA1*	c.6531‐12G > A	NA	Intron 45	Likely benign*	Htz	intronic	No			
HS12	*SLC4A1*	c.2386G > A	p.(Gly796Arg)	Exon 18	VUS	Htz	missense	Yes	38.4	120	0.598
	*SPTA1*	c.775G > A	p.(Ala259Thr)	Exon 6	VUS	Htz	missense	No			
HS13	*SLC4A1*	c.118G > A	p.(Glu40Lys)	Exon 4	Likely benign*	Htz	missense	No	34.6	167	0.613
	*SPTA1*	c.775G > A	p.(Ala259Thr)	Exon 6	VUS	Htz	missense	No			
HS15	*SPTB*	c.3764+1G > A	Splice site	Intron 16	Likely pathogenic	Htz	splice	Yes	52.1	177	0.553
	*SPTA1*	c.2909C > A	p.(Ala970Asp)	Exon 21	Likely benign*	Htz	missense	No			
HS16	*SPTB*	c.3764+1G > A	Splice site	Intron 16	Likely pathogenic	Htz	splice	Yes	48.5	185	0.538
HS17	*SPTB*	Large_del	NA	Exons 2–3	Likely pathogenic	Htz	frameshift	Yes	55.3	18	0.533
	*SPTA1*	c.6531‐12G > A	NA	Intron 45	Likely benign*	Hmz	intronic	No			
HS18	*SLC4A1*	c.733G > A	p.(Val245Met)	Exon 9	VUS	Htz	missense	No	49.2	185	0.556
	*ANK1*	c.4915_4921del***CACGAGT	p.(His1639Glyfs*90)	Exon 39	Likely pathogenic	Htz	frameshift	Yes			
	*SPTA1*	c.6531‐12G > A	NA	Intron 45	Likely benign*	Hmz	intronic	No			
HS19	*SLC4A1*	c.118G > A	p.(Glu40Lys)	Exon 4	Likely benign*	Htz	missense	No	62.5	199	0.387
	*SPTA1*	c.2909C > A	p.(Ala970Asp)	Exon 21	Likely benign*	Htz	missense	No			
	*SPTA1*	c.2671C > T	p.(Arg891*)	Exon 19	Likely pathogenic	Htz	nonsense	No			
	*SPTA1*	c.4347G > T	p.(Lys1449Asn)	Exon 31	VUS	Htz	missense	No			
	*SPTA1*	c.4339‐99C > T	NA	Intron 30	Likely benign*	Htz	intronic	No			
HS20	*SLC4A1*	c.2057+1G > A	Splice site	Intron 16	Likely pathogenic	Htz	splice	Yes	53.6	189	0.578
	*SLC4A1*	c.2057+5G > A	NA	Intron 16	VUS	Htz	intronic	Yes			
HS21	*SPTA1*	c.2909C > A	p.(Ala970Asp)	Exon 21	Likely benign*	Htz	missense	No	41.3	168	0.613
	*SPTA1*	c.6531‐12G > A	NA	Intron 45	Likely benign*	Htz	intronic	No			
HS22	*EPB42*	c.2054C > T	p.(Thr685Met)	Exon 13	VUS	Htz	missense	No	40.8	178	0.597
	*SPTA1*	c.6531‐12G > A	NA	Intron 45	Likely benign*	Hmz	intronic	No			
HS23	*SPTB*	Large_del	NA	Exons 2–3	Likely pathogenic	Htz	frameshift	Yes	48.5	207	0.485
	*SPTA1*	c.6531‐12G > A	NA	Intron 45	Likely benign*	Htz	intronic	No			
HS25	*SPTB*	c.2431_2450del***GAAGAGTTTCG***GGATTCCCC	p.(Glu811Argfs*44)	Exon 13	Likely pathogenic	Htz	frameshift	Yes	36.2	166	0.578
	*SPTA1*	c.6531‐12G > A	NA	Intron 45	Likely benign*	Htz	intronic	No			
HS26	*SPTB*	c.2431_2450del***GAAGAGTTTCG***GGATTCCCC	p.(Glu811Argfs*44)	Exon 13	Likely pathogenic	Htz	frameshift	Yes	51.8	185	0.491
	*SPTA1*	c.6531‐12G > A	NA	Intron 45	Likely benign*	Htz	intronic	No			
HS27	*SPTB*	c.2588G > A	p.(Trp863*)	Exon 13	Likely pathogenic	Htz	nonsense	Yes	56.1	180	0.444
HS28	*SPTB*	c.2588G > A	p.(Trp863*)	Exon 13	Likely pathogenic	Htz	nonsense	Yes	53.6	176	0.506
HS29	*SPTA1*	c.2320C > T	p.(Arg774*)	Exon 17	Likely pathogenic	Htz	nonsense	No	38.3	174	0.597
	*SPTA1*	c.6531‐12G > A	NA	Intron 45	Likely benign*	Hmz	intronic	No			
HS30	*SPTA1*	c.2909C > A	p.(Ala970Asp)	Exon 21	Pathogenic	Hmz	missense	No	37.0	185	0.581
	*SPTA1*	c.7068A > C	p.(Glu2356Asp)	Exon 51	VUS	Htz	missense	No			
	*SPTA1*	c.3940T > C	p.(Ser1314Pro)	Exon 28	VUS	Htz	missense	No			
	*SPTA1*	c.4339‐99C > T	NA	Intron 30	Likely benign*	Htz	intronic	No			
	*SPTA1*	c.6531‐12G > A	NA	Intron 45	Likely benign*	Htz	intronic	No			
HS31	*SPTA1*	c.6531‐12G > A	NA	Intron 45	Likely benign*	Htz	intronic	No	37.4	152	0.602
HS32	*SPTA1*	c.6531‐12G > A	NA	Intron 45	Likely benign*	Htz	intronic	No	32.9	154	0.623
HS33	*SLC4A1*	c.118G > A	p.(Glu40Lys)	Exon 4	Likely benign*	Htz	missense	No	31.7	179	0.604
HS34	*EPB41*	c.820C > T	p.(Gln274*)	Exon 5	Likely pathogenic	Htz	nonsense	Yes	33.3	171	0.556
HS35	*SPTA1*	c.6531‐12G > A	NA	Intron 45	Likely benign*	Hmz	intronic	No	31.7	162	0.608
HS36	*SPTA1*	c.6531‐12G > A	NA	Intron 45	Likely benign*	Htz	intronic	No	30.3	185	0.607
HS37	*SPTA1*	c.6531‐12G > A	NA	Intron 45	Likely benign*	Hmz	intronic	No	34.4	162	0.607
HS38	*ANK1*	c.2858+1G > T	Splice site	Intron 26	Likely pathogenic	Htz	splice	Yes	53.9	185	0.493
	*SPTA1*	c.6531‐12G > A	NA	Intron 45	Likely benign*	Htz	intronic	No			
HS39	*SPTA1*	c.1599+1G > T	Splice site	Intron 12	Likely pathogenic	Htz	splice	Yes	42.0	167	0.576
	*SPTA1*	c.1450G > A	p.(Asp484Asn)	Exon 11	VUS	Htz	missense	Yes			
	*SPTA1*	c.6531‐12G > A	NA	Intron 45	Likely benign*	Hmz	intronic	No			
HS40	*SPTA1*	c.6531‐12G > A	NA	Intron 45	Likely benign*	Htz	intronic	No	24.9	170	0.609
HS43	*SPTA1*	c.2909C > A	p.(Ala970Asp)	Exon 21	Likely benign*	Htz	missense	No	10.0	165	0.598
HS44	*SPTA1*	c.2909C > A	p.(Ala970Asp)	Exon 21	Likely benign*	Htz	missense	No	36.7	165	0.618
HS45	*SPTA1*	c.6531‐12G > A	NA	Intron 45	Likely benign*	Htz	intronic	No	37.0	160	0.617
HS46	*SPTB*	c.5266C > T	p.(Arg1756*)	Exon 25	Pathogenic	Htz	nonsense	No	40.9	185	0.532
HS51	*SLC4A1*	c.2021T > G	p.(Val674Gly)	Exon 16	VUS	Htz	missense	Yes	40.5	165	0.608
	*SPTA1*	c.6531‐12G > A	NA	Intron 45	Likely benign*	Hmz	intronic	No			
HS52	*ANK1*	c.1486G > A	p.(Val496Ile)	Exon 13	VUS	Htz	missense	No	34.1	142	0.615
HS53	*SPTB*	c.4891C > T	p.(Arg1631Cys)	Exon 23	VUS	Htz	missense	No	33.4	173	0.599
HS54	*SPTA1*	c.2909C > A	p.(Ala970Asp)	Exon 21	Likely benign*	Hmz	missense	No	40.0	188	0.554
	*SPTA1*	c.4347G > T	p.(Lys1449Asn)	Exon 31	VUS	Htz	missense	No			
	*SPTA1*	c.4339‐99C > T	NA	Intron 30	Likely benign*	Hmz	intronic	No			
HS55	*SPTB*	c.3479G > A	p.(Arg1160His)	Exon 15	VUS	Htz	missense	No	35.4	160	0.604
	*SPTA1*	c.6531‐12G > A	NA	Intron 45	Likely benign*	Htz	intronic	No			
HS56	*SPTA1*	c.6531‐12G > A	NA	Intron 45	Likely benign*	Htz	intronic	No	24.5	164	0.609
HS57	*SPTA1*	c.6531‐12G > A	NA	Intron 45	Likely benign*	Htz	intronic	No	25.3	172	0.623
HS59	*ANK1*	c.127‐39554G > A	NA	Promoter 5`UTR/Intron 1	Likely benign*	Htz	intronic	No	30.2	171	0.618
	*ANK1*	c.127‐39509T > C	NA	Promoter 5`UTR/Intron 1	Likely benign*	Htz	intronic	No			
	*ANK1*	c.5302C > A	p.(Gln1768Lys)	Exon 40	VUS	Htz	missense	Yes			
HS60	*SPTA1*	c.6896G > T	p.(Cys2299Phe)	Exon 50	VUS	Htz	missense	No	34.8	166	0.606
	*EPB41*	c.1700G > A	p.(Gly567Asp)	Exon 12	VUS	Htz	missense	No			
HS61	*SPTA1*	c.6531‐12G > A	NA	Intron 45	Likely benign*	Htz	intronic	No	21.6	161	0.608
HS62	*SPTA1*	c.6531‐12G > A	NA	Intron 45	Likely benign*	Htz	intronic	No	32.6	158	0.612
HS65	*SPTB*	c.3496C > T	p.(Gln1166*)	Exon 15	Likely pathogenic	Htz	nonsense	Yes	49.5	194	0.544
	*SPTA1*	c.4605+4delA	NA	Intron 32	VUS	Htz	intronic	No			
HS66	*SPTB*	c.5860A > G	p.(Thr1954Ala)	Exon 27	VUS	Htz	missense	Yes	38.3	147	0.597
	*SPTA1*	c.2909C > A	p.(Ala970Asp)	Exon 21	Likely benign*	Htz	missense	No			
	*SPTB*	c.5032G > C	p.(Val1678Leu)	Exon 24	VUS	Htz	missense	Yes			
HS68	*SPTA1*	c.6531‐12G > A	NA	Intron 45	Likely benign*	Htz	intronic	No	38.0	160	0.601
HS69	*SPTB*	c.26A > C	p.(Asn9Thr)	Exon 1	VUS	Htz	missense	No	36.0	154	0.619
HS70	*SPTA1*	c.6531‐12G > A	NA	Intron 45	Likely benign*	Hmz	intronic	No	56.3	165	0.524
HS71	*SPTA1*	c.6531‐12G > A	NA	Intron 45	Likely benign*	Htz	intronic	No	36.9	167	0.592
HS72	*SPTA1*	c.1112+1G > T	Splice site	Intron 8	Likely pathogenic	Htz	splice	Yes	33.4	178	0.571
	*SPTA1*	c.6531‐12G > A	NA	Intron 45	Likely benign*	Hmz	intronic	No			
HS73	*SPTA1*	c.6531‐12G > A	NA	Intron 45	Likely benign*	Htz	intronic	No	19.1	196	0.613
HS75	*SPTA1*	c.6531‐12G > A	NA	Intron 45	Likely benign*	Htz	intronic	No	38.7	158	0.615
HS78	*SPTA1*	c.2464+1G > A	Splice site	Intron 17	Likely pathogenic	Htz	splice	No	37.5	179	0.579
	*SPTA1*	c.6531‐12G > A	NA	Intron 45	Likely benign*	Htz	intronic	No			
HS80	*SPTB*	c.398T > G	p.(Met133Arg)	Exon 3	VUS	Htz	missense	Yes	31.0	159	0.594
	*SPTB*	c.6856G > A	p.(Ala2286Thr)	Exon 35	VUS	Htz	missense	No			
HS81	*SLC4A1*	c.2701C > T	p.(Arg901Trp)	Exon 20	VUS	Htz	missense	No	33.4	160	0.600
	*SPTA1*	c.2909C > A	p.(Ala970Asp)	Exon 21	Likely benign*	Htz	missense	No			
	*SLC4A1*	c.1162C > T	p.(Arg388Cys)	Exon 11	VUS	Htz	missense	No			
	*SPTA1*	c.4339‐99C > T	NA	Intron 30	Likely benign*	Htz	intronic	No			
HS84	*EPB42*	c.1477G > A	p.(Gly493Ser)	Exon 10	VUS	Htz	missense	No	24.0	145	0.617
	*SPTA1*	c.6531‐12G > A	NA	Intron 45	Likely benign*	Htz	intronic	No			
HS85	*EPB41*	c.1700G > A	p.(Gly567Asp)	Exon 12	VUS	Hmz	missense	No	51.0	173	0.517
	*EPB42*	c.826C > T	p.(Arg276Trp)	Exon 6	VUS	Htz	missense	No			
HS86	*SPTB*	c.379C > T	p.(Arg127Cys)	Exon 3	VUS	Htz	missense	No	36.2	180	0.579
	*ANK1*	c.3571C > T	p.(Pro1191Ser)	Exon 30	VUS	Htz	missense	Yes			
HS87	*SPTB*	c.1134_1135delGA	p.(Lys379Serfs* 12)	Exon 9	Likely pathogenic	Htz	frameshift	Yes	52.3	193	0.508
HS88	*ANK1*	c.3173G > A	p.(Trp1058*)	Exon 28	Likely pathogenic	Htz	nonsense	Yes	48.1	190	0.541
	*ANK1*	c.38A > T	p.(Asp13Val)	Exon 1	VUS	Htz	missense	Yes			
	*SPTA1*	c.6531‐12G > A	NA	Intron 45	Likely benign*	Htz	intronic	No			
HS89	*SLC4A1*	c.2102G > A	p.(Gly701Asp)	Exon 17	Pathogenic^†^	Htz	missense	No	49.0	160	0.561
	*SPTB*	c.6626T > C	p.(Val2209Ala)	Exon 33	VUS	Htz	missense	Yes			
	*SLC4A1*	c.92T > C	p.(Met31Thr)	Exon 3	VUS	Htz	missense	No			
HS90	*SPTA1*	c.4564A > G	p.(Thr1522Ala)	Exon 32	VUS	Htz	missense	No	33.0	157	0.590
	*ANK1*	c.127‐39554G > A	NA	Promoter 5`UTR/Intron 1	Likely benign*	Htz	intronic	No			
	*ANK1*	c.127‐39509T > C	NA	Promoter 5`UTR/Intron 1	Likely benign*	Htz	intronic	No			
HS91	*ANK1*	c.127‐39554G > A	NA	Promoter 5`UTR/Intron 1	Likely benign*	Htz	intronic	No	43.0	194	0.555
	*ANK1*	c.127‐39509T > C	NA	Promoter 5`UTR/Intron 1	Likely benign*	Htz	intronic	No			
	*ANK1*	c.542T > C	p.(Leu181Pro)	Exon 6	VUS	Htz	missense	Yes			
	*SPTA1*	c.6531‐12G > A	NA	Intron 45	Likely benign*	Htz	intronic	No			
HS92	*ANK1*	c.491T > C	p.(Leu164Pro)	Exon 5	VUS	Htz	missense	Yes	44.0	173	0.567
	*SPTA1*	c.6531‐12G > A	NA	Intron 45	Likely benign*	Htz	intronic	No			
HS93	*SPTA1*	c.6531‐12G > A	NA	Intron 45	Likely benign*	Htz	intronic	No	39.5	160	0.604
HS94	*SPTA1*	c.1112+1G > T	Splice site	Intron 8	Likely pathogenic	Htz	splice	Yes	34.7	175	0.568
	*SPTA1*	c.6531‐12G > A	NA	Intron 45	Likely benign*	Hmz	intronic	No			
HS95	*SLC4A1*	c.443A > G	p.(Gln148Arg)	Exon 6	VUS	Htz	missense	Yes	46.0	177	0.579
	*SPTA1*	c.6531‐12G > A	NA	Intron 45	Likely benign*	Htz	intronic	No			
HS96	*SPTA1*	c.6531‐12G > A	NA	Intron 45	Likely benign*	Htz	intronic	No	34.6	163	0.604
HS97	*ANK1*	c.3508A > T	p.(Ser1170Cys)	Exon 30	VUS	Htz	missense	Yes	39.8	167	0.600
HS98	*SLC4A1*	c.1564G > A	p.(Glu522Lys)	Exon 13	VUS	Htz	missense	Yes	51.1	185	0.560
HS99	*SPTA1*	c.2909C > A	p.(Ala970Asp)	Exon 21	Likely benign*	Htz	missense	No	41.1	201	0.511
	*SPTA1*	c.3792_3793dupAA	p.(Met1265Lysfs* 4)	Exon 27	Likely pathogenic	Htz	frameshift	Yes			
	*SPTA1*	c.4339‐99C > T	NA	Intron 30	Likely benign*	Htz	intronic	No			

* Indicated in Illumina as likely benign but may be pathogenic in homozygous or compound heterozygous state (*STPA1*:c.6531‐12G > A is only potentially pathogenic in compound heterozygotes).

^†^ One mutation in HS89 (*SCL4A1*:c.2102 G > A) was described as pathogenic in Illumina. This pathogenicity is related to distal tubular renal acidosis and was interpreted as VUS.

Abbreviations: EImax, elongation index maximum; Hmz, homozygous; Htz, hetetozygous; NA, not applicable; UTR, untranslated region; VUS, variant of uncertain significance.

Seventeen of the 58 individuals harbored more than one mutation. In total, 34 individuals harbored one or more (likely) pathogenic variants and 24 individuals harbored one or more VUS as the only mutations (Figure 1). Forty‐one individuals had no proven mutations. One *SLC4A1*:c.2102G > A mutation (HS89) associated with renal tubular acidosis and renal membranopathy was classified as pathogenic. In the context of HS, this mutation was interpreted as a VUS. *SPTA1* is associated with autosomal recessive HS [23]. To simplify our efforts to set a diagnostic threshold for the EMA binding test, we considered individuals with only (likely) pathogenic *SPTA1* mutation(s) as having 'VUS'.

### 3.3 EMA binding test and osmotic gradient ektacytometry as predictors of mutation status

**FIGURE 2:**
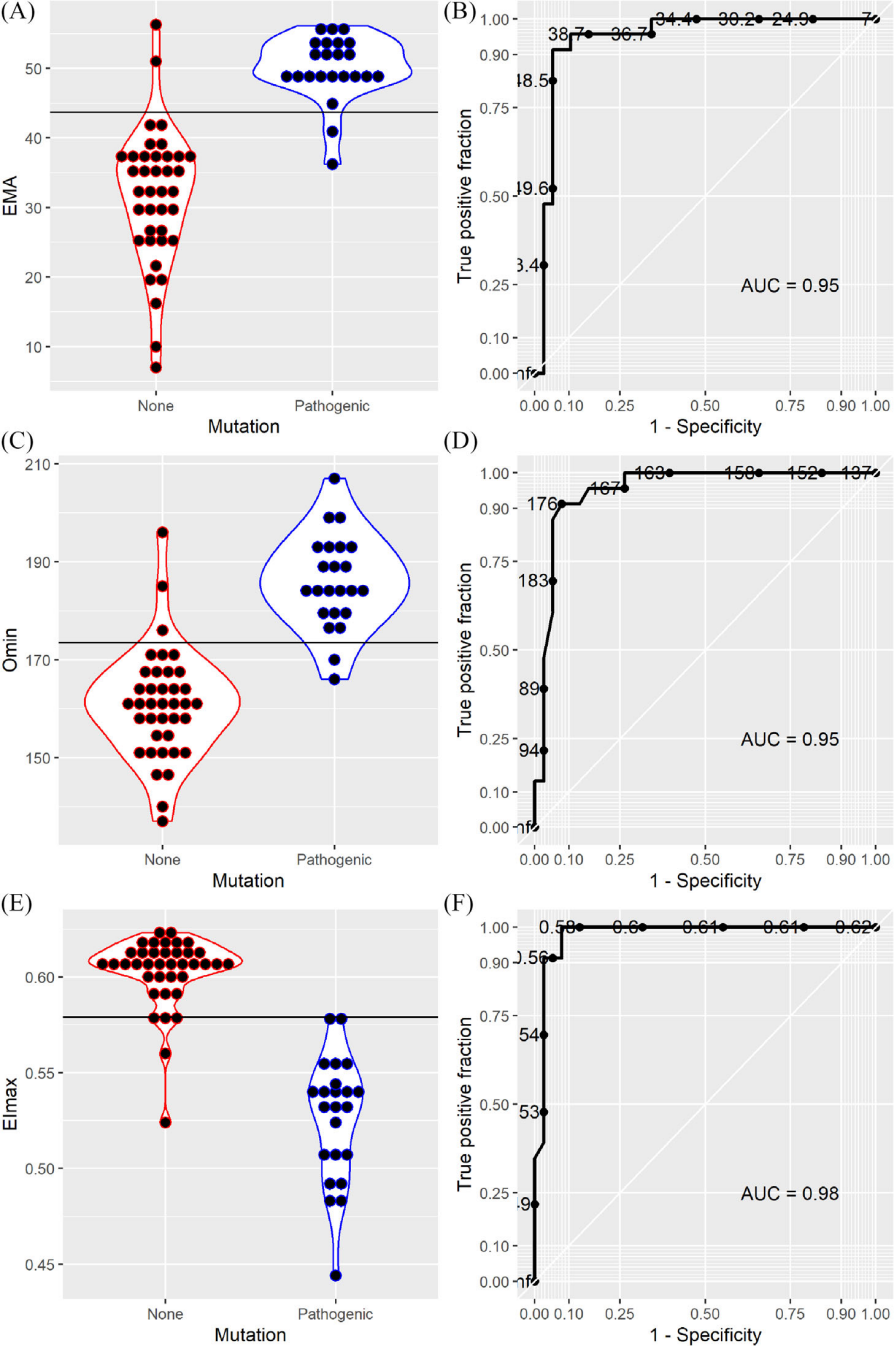
**Evaluation of modified EMA binding test and ektacytometry against mutational status**. Individual distribution of ΔMFI% values (EMA) and the associated ROC curve (A‐B), Omin values and the associated ROC curve (C‐D), EImax values and the associated ROC curve (E‐F), in 95 individuals with suspected hereditary spherocytosis. Thirty‐eight individuals had no proven mutations in red blood cell cytoskeleton protein genes, 33 had one or more (likely) pathogenic variants (excluding *EPB41*), and 34 had only *SPTA1* mutations or variants of uncertain significance (VUS; not shown)

We demonstrated significantly higher EMA (ΔMFI%) values in individuals with (likely) pathogenic variants compared to individuals without mutations. Similarly, EMA values were significantly higher in individuals with VUS compared to individuals with no mutations (Figure 2A; *p *= 0.00044). In ROC curve analysis, we found that a threshold of ≥43.6 was optimal for discriminating between individuals with (likely) pathogenic variants and individuals with no mutations (AUC = 95%) (Figure 2B).

O_min_ values were significantly higher in individuals with (likely) pathogenic variants compared to individuals without mutations. This corresponds to an increased osmotic fragility in individuals with (likely) pathogenic variants. A significant difference between O_min_ values in individuals with VUS and individuals with no underlying mutations was also observed, although to a much lesser extent (Figure 2C; *p = *0.0023). In our ROC curve analysis, we found that a threshold of 174 mOsm/kg was optimal for discriminating between individuals with (likely) pathogenic variants and individuals without mutations (Figure 2D; AUC = 95%).

When comparing EI_max_ between individuals with (likely) pathogenic variants and individuals without mutations, values were significantly lower in the subgroup with pathogenic mutations, indicating reduced RBC deformability (Figure 2E). When comparing EI_max_ values from individuals with VUS and individuals without mutations, the difference was still significant (Figure 2E; *p <* 0.0002). Our ROC curve analysis demonstrated an optimal threshold of <0.579 for discriminating individuals with (likely) pathogenic variants from individuals without mutations (Figure 2F; AUC = 98%).

Applying these thresholds, we subsequently calculated: sensitivity, specificity, positive predictive value (PPV), negative predictive value (NPV) and accuracy for the individual parameters (Table 2). All demonstrated sensitivities, specificities, NPV and PPV above 87%. As a single parameter, EI_max_, yielded the best results with an accuracy of 95.1%.

**TABLE 2:** Sensitivity, Specificity, positive predictive value (PPV), negative predictive value (NPV) and accuracy of the EMA binding test and osmotic gradient ektacytometry (Omin and EImax) in 95 patients with suspected hereditary spherocytosis

	Sensitivity (%)	Specificity (%)	PPV (%)	NPV (%)	Accuracy (%)
EMA ≥ 43.6	91.3	94.7	91.3	94.7	93.4
Omin ≥ 174	91.3	92.1	87.5	94.6	91.8
EImax < 0.579	100	92.1	88.5	100	95.1
Omin ≥ 166 EImax < 0.579	100	97.4	95.8	100	98.7
Omin ≥ 166 EImax < 0.579 EMA ≥ 43.6	91.3	97.4	95.5	94.9	94.3

Results are given for each parameter individually and in combination.

Abbreviations: EImax, elongation index maximum; EMA, eosin‐5′‐maleimide; NPV, negative predictive value; PPV, positive predictive value.

### 3.4 Combining osmotic gradient ektacytometry and the EMA binding test to predict mutation status

**FIGURE 3:**
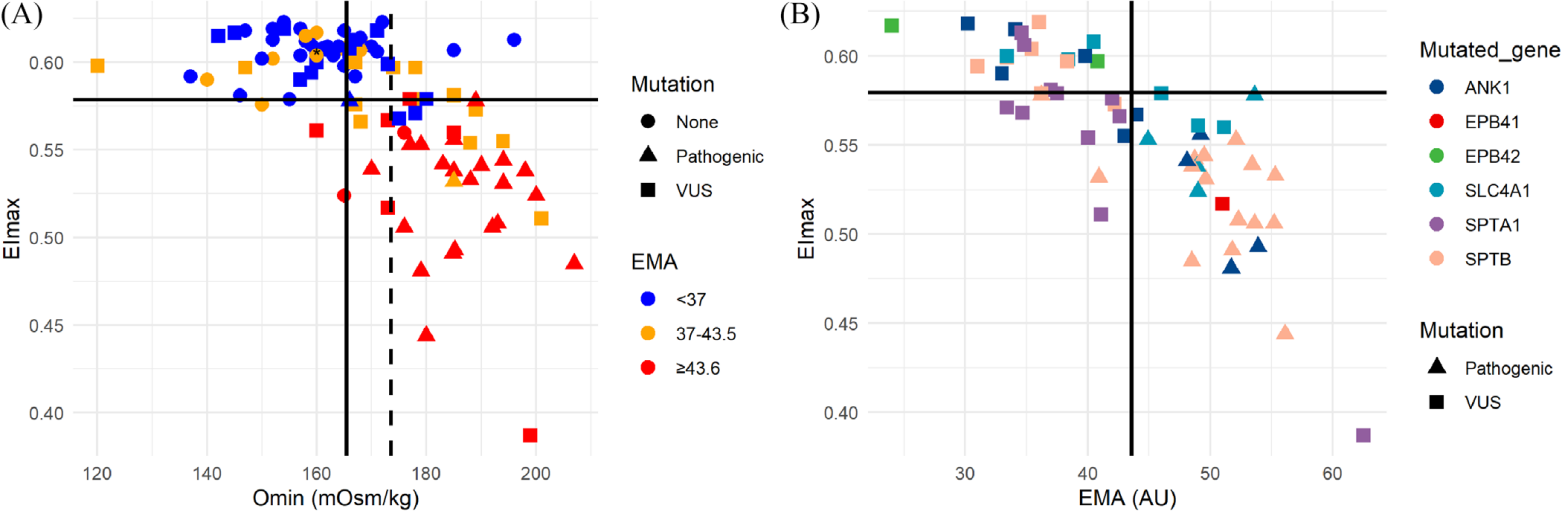
**Genetic variants and functional testing**. (A) Distribution and relationship between the EMA binding test, Omin and EImax in 95 individuals with suspected hereditary spherocytosis. Forty‐one individuals had no proven mutations in red blood cell cytoskeleton protein genes, 33 had one or more (likely) pathogenic variants and 34 had only SPTA1 mutations or variants of uncertain significance (VUS). Full lines correspond to Omin 166 mOsm/kg and EImax 0.579. Dotted line corresponds to Omin 174 mOsm/kg. *Represents two individuals with similar Omin and EImax. (B) Distribution and relationship between the EMA binding test, EImax and mutations in 57 individuals suspected of having hereditary spherocytosis. Thirty‐three individuals had one or more (likely) pathogenic mutations (excluding *EPB41*), and 34 had only *SPTA1* mutations or variants of uncertain significance (VUS)

The distribution of all 95 samples, based on the EMA binding test, O_min_ and EI_max_ values, is illustrated in Figure 3.

When combining the calculated thresholds for O_min_ and EI_max_ (O_min_ ≥ 174 mOsm/kg and EI_max_ < 0.579), 21 of 23 individuals with (likely) pathogenic variants were double positive (i.e. true positive), and two were false negative (Figure 3A). In addition, one individual without (likely) pathogenic variants remained double positive (Figure 3). Adjusting the O_min_ ≥ 166 mOsm/kg while keeping EI_max_ < 0.579 improved the sensitivity to 100%, while keeping an excellent specificity of 97.4% (Table 2). Subsequently, we calculated the sensitivity, specificity, PPV, NPV and accuracy using the obtained EMA, O_min_, and EI_max_ thresholds in combination. However, this approach resulted in a marked reduction of sensitivity and NPV without improving other measures (Table 2).

Figure 3B shows an excellent relationship between the modified EMA binding test with fluorescent beads and EI_max_ in individuals with (likely) pathogenic variants and VUS, regardless of the type of the mutated gene.

## 4 DISCUSSION

In this study, we assessed the number of RBC cytoskeleton protein gene mutations in a population of individuals with suspected HS, using pathogenic mutations as the gold standard. This enabled us to set a diagnostic cut‐off value for our newly described modified EMA binding test with fluorescent beads (Figure 2 and Table 2), thereby alleviating it from the otherwise obligatory use of up to six healthy control samples [5]. Using the EMA binding test alone, we obtained a diagnostic accuracy (Table 2) comparable to those previously reported using healthy controls [44, 45, 46, 47]. In many settings, obtaining suitable control samples can be challenging [8]. Furthermore, the inherent variation in control samples complicates interlaboratory comparisons and quality assessment schemes [24]. Our approach has demonstrated a robust performance, comparable to that of the traditional EMA‐binding test with healthy controls [8] and osmotic gradient ektacytometry (the gold standard of membranopathy diagnostics) across a range of causative genes (Figure 4).

As such, this study differs from previous studies in which HS has typically been defined by clinical phenotype or sulphate polyacrylamide gel electrophoresis (SDS‐PAGE) [1, 28, 32, 40, 47, 48]. We are aware that laboratory screening tests must be related to clinical phenotype. Although we did not have access to patient records and clinical data, we must assume that individuals referred to our laboratory on suspicion of HS had clinical symptoms consistent with the disease. All individuals with true HS are expected to have one or more underlying pathogenic germline mutations, although they are not always identified [16]. Using genotype as the gold standard for HS diagnosis when making an ROC analysis for the EMA‐binding test could eliminate confirmation bias. It is likely that some individuals harbor mutations or deletions not detected in the applied tNGS panel and, consequently, remain undiagnosed [16, 28, 32], but this should not have significant impact on the ROC analysis determining the EMA‐binding test cut‐off value. Furthermore, congenital dyserythropoietic anaemia type II often mimics HS on the EMA binding test and osmotic gradient ektacytometry [34, 35], but the causative gene *SEC23B* was not included in our tNGS panel. As a diagnostic laboratory, we did not have access to data or samples from relatives, which prevented determination of inheritance patterns.

Excluding *EPB41*, we detected a total of 76 underlying (likely) pathogenic variants and VUS (Table 1), 42 previously undescribed. They were found in *SPTB *> *SPTA1* > *SLC4A1* > *ANK1* > *EPB42* (listed according to mutation frequency). In previous studies, defect or lack of ankyrin has often been reported as the most frequent mutation in HS, particularly in Northern Europe [2, 49, 50]. The order of affected genes in our study differs from those seen in studies in which clinical features and non‐DNA‐based diagnostics define the disease. The high number of *SPTA1* mutations found in our population reflects inclusion of the common hypomorph variants such as c.4339‐99C > T (α‐spectrin^LEPRA^), which in its heterozygous form should not cause overt haemolysis [51].

In our study, 32 of 34 individuals with detected (likely) pathogenic variants had heterozygous non‐missense mutations, and two individuals had homozygous *SPTA1* missense mutations: c.2909 C > A (α‐spectrin^Bug Hill^; HS30 and HS54 in Table 1). c.2909 C > A was originally classified as pathogenic (autosomal recessive) [52], but this is likely due to frequent co‐occurrence of c.4339‐99C > T in *cis* [51]. In contrast, all VUS were missense mutations, except three intron mutations (two in the *SPTA1* gene and one in the *SLC4A1* gene).

As α‐spectrin is synthesised in excess [53], heterozygous *SPTA1* pathogenic mutations are considered clinically benign but may be pathogenic in homozygous and compound heterozygous state. Accordingly, individuals heterozygous for (likely) pathogenic *STPA1* mutations were not used in ROC analysis but several had borderline ΔMFI% changes (Table 1). This is in line with some degree of RBC surface area loss and even mild clinical haemolysis as previously described [28]. Four individuals in our study only harbored a heterozygous *SPTA1* mutation (HS29, HS72, HS78, HS94 in Table 1), and one was homozygous for two common missense mutation in *SPTA1*: α‐spectrin^LEPRA^ and α‐spectrin^Bug Hill^ combined with two VUS (HS30). None of these exceeded the EMA cut‐off value. The remaining five individuals with *SPTA1* mutations all had EMA values ≥ 40. Five of these had multiple *SPTA1* mutations: 3 α‐spectrin^LEPRA^ combined with truncating mutations (HS19, HS99) or with a homozygous *SPTA1* missense mutation (HS54), one individual with a pathogenic splice‐site mutation combined with 2 VUS interpreted as compound heterozygous (HS7), and one individual with a splice site mutation as the only alteration (*SPTA1*:c.1599+1G > T)(HS39). The prevalence of α‐spectrin^LELY^ was notably high (43%) compared to studies of the background population [54].

Although all 10 individuals with pathogenic *SPTA1* mutations were double positive on ektacytometry (O_min_ ≥ 166 mOsm/kg and EI_max_ < 0.579), it is worth noting that five of these were not detected by the EMA‐binding test applied. Positive ektacytometry in these 10 individuals likely suggests that pathogenic mutations functionally decrease the stability of the RBC cytoskeleton.

As single parameters, results were comparable for O_min_ and the EMA binding test, whereas EI_max_ was superior compared to both, when separating individuals with (likely) pathogenic variants from those without mutations (Table 2). Combining O_min_ and EI_max_ provided an excellent accuracy of 98.7% and adding EMA on top provided no benefit (Table 2).

Our accuracy measures may not be entirely representative, as we discarded individuals with homozygous *SPTA1* mutations and VUS. Likely, several of these have HS as evaluated by their EMA binding test and ektacytometry (Table 1). In contrast, some causative mutations and deletions could have been missed by our tNGS approach. Furthermore, our study was not powered to calibrate the EMA threshold for the individual mutated genes. In this study, however, we aimed to set a diagnostic threshold for the EMA binding test without using healthy controls – and as such not to determine its precise accuracy. For this purpose, these limitations are accep table in our opinion.

In conclusion, our data demonstrate the reliability of the modified EMA binding test with rainbow beads when defining a cut‐off for HS by mutational status. When established, this approach makes the test more manageable and less time‐consuming. Ensuring consistency of data over time requires careful evaluation of new batches of rainbow beads and EMA dye. Interlaboratory work is ongoing to test whether this novel approach can be applied in a multicenter setting.

## FUNDING INFORMATION

This study was supported by funding from the Department of Hematology, Rigshospitalet, Copenhagen, Denmark.

## CONFLICT OF INTEREST

Andreas Glenthøj: Agios, bluebird bio, Bristol Myers Squibb, Novartis: consultancy. Research grant: Alexion, Saniona. Honoraria: Novo Nordisk. The authors declare no conflict of interest relevant to the manuscript.

## AUTHOR CONTRIBUTIONS

Henrik Birgens, Jesper Petersen and Andreas Glenthøj planned this study. Jesper Petersen performed all tNGS and ektacytometry analyses. Jesper Petersen, Andreas Glenthøj and Christian Brieghel performed the statistical analysis. Henrik Birgens, Jesper Petersen, Christian Brieghel, Andreas Glenthøj, Amina Nardo‐Marino and Richard van Wijk analysed data and wrote the manuscript. Andreas Glenthøj prepared all figures. All authors contributed to the final approved version of this report.

## Data Availability

The data that support the findings of this study are available from the corresponding author upon reasonable request.
